# The causal relationship between gut microbiota and lower extremity deep vein thrombosis combined with pulmonary embolism

**DOI:** 10.3389/fmicb.2024.1301737

**Published:** 2024-10-02

**Authors:** Qiyang Xu, Jihong Fang, Yi Wang, Dehai Lang, Bin Xu

**Affiliations:** ^1^Department of General Surgery, Ningbo No.2 Hospital, Ningbo, China; ^2^Department of Emergency, Ningbo Medical Center Lihuili Hospital, The Lihuili Affiliated Hospital of Ningbo University, Ningbo, China; ^3^Department of Radiotherapy and Chemotherapy, Ningbo No.2 Hospital, Ningbo, China

**Keywords:** gut microbiota, deep vein thrombosis, pulmonary embolism, Mendelian randomization, genetic

## Abstract

**Background:**

Over the years, numerous studies have explored the relationship between gut microbiota and lower extremity deep vein thrombosis (LEDVT) and pulmonary embolism (PE). The present study utilized Mendelian randomization (MR) to assess the causal link between gut microbiota and LEDVT combined with PE.

**Methods:**

Human gut microbiota genome-wide association study (GWAS) summary data from the MiBioGen consortium (*n* = 18,340) were utilized. Summary-level data on LEDVT (2,116 cases and 359,078 controls) and LEDVT combined with PE (4,319 cases and 356,875 controls) were obtained from the IEU Open GWAS project. MR analysis was conducted using the inverse variance weighted (IVW) method as the primary analysis. Additionally, MR-Egger, weighted median, weighted mode, and simple mode were employed as supplementary methods. Sensitivity analyses, including tests for heterogeneity and horizontal pleiotropy, were performed. Lastly, reverse MR analysis was performed.

**Results:**

The IVW analyses revealed seven causal relationships between genetic liability in the gut microbiota and LEDVT and five causal relationships between genetic liability in the gut microbiota and LEDVT combined with PE. The intersection of these outcomes identified that the genus *Butyricicoccus* reduced the risk of both LEDVT and LEDVT combined with PE, while the genus *Clostridium innocuum* increased the risk for both conditions.

**Conclusion:**

This study demonstrates that the gut microbiota is causally associated with LEDVT and LEDVT combined with PE. Our findings provide valuable insights into the underlying mechanisms and suggest potential avenues for further clinical investigations of these conditions.

## 1 Introduction

Venous thromboembolism (VTE), including deep vein thrombosis (DVT) and PE (Konstantinides et al., 2020), is the third most common acute cardiovascular disease in the world (Raskob et al., 2014). According to literature reports, the estimated incidence rates of PE range from 39 to 115 cases per 100,000 (Cheuk et al., 2004; Centers for Disease Control and Prevention (CDC), 2012), while the estimated incidence rates of DVT range from 53.1 to 162 per 100,000 (Jang et al., 2011; Nordström et al., 1992). LEDVT is the most common form of DVT (Stubbs et al., 2018), and if left untreated, 30–50% of patients with LEDVT eventually develop PE (Anderson et al., 1991). It is now understood that 90% of pulmonary embolisms are caused by LEDVT (Kruit et al., 1991), when both LEDVT and PE are present, PE is almost always caused by LEDVT. Current evidence suggests that the mortality rate of PE is high, with an estimated 126,145 (34%) sudden fatal cases in six European countries with a total population of 454.4 million (Cohen et al., 2007). VTE also has a significant economic burden, with annual costs ranging from 2 to 10 billion in the United States (Park et al., 2009). VTE is a preventable condition associated with genetic and acquired risk factors. Genetic risk factors, such as the factor V Leiden variant (Simone et al., 2013) and prothrombin F2 gene variant G20210A (Morange et al., 2015), play a crucial role in the development of VTE. Therefore, a comprehensive understanding of the genetic factors associated with DVT and PE is essential for advancing future research in this area.

The gut microbiota is a complex community of microorganisms residing in the digestive system, consisting primarily of bacteria but also viruses and fungi (Turnbaugh et al., 2007). It is vital in regulating human health and diseases (Heintz-Buschart and Wilmes, 2018). The composition of the gut microbiota is influenced by various factors, including dietary choices (David et al., 2014) and genetic elements within the host (Bonder et al., 2016). Several studies have found that the gut microbiota composition can modulate the risk of VTE. For example, antibiotics have been shown to modulate the gut microbiome and inhibit pulmonary thrombosis in mice (Zhang et al., 2023). Gut microbiota metabolites, such as trimethylamine N-oxides (TMAO), may also contribute to VTE risk and recurrence (Fraser et al., 2020). Gut dysbiosis can lead to the translocation of lipopolysaccharides (LPS) into the systemic circulation, causing inflammation and affecting coagulation and platelet activation (Violi et al., 2023). The Gram-negative transition of gut microbes has been associated with systemic inflammation and increased FVIII activity, contributing to VTE development (Grimnes et al., 2022). A recent study revealed that intestinal microbiota colonization restored the production of von Willebrand factor in hepatic endothelial cells and prevented platelet aggregation in Toll-like receptor 2 (TLR2) deficient mice, thereby correcting plasma vWF levels and thrombus growth in TLR2 deficient mice (Jäckel et al., 2017). In another study, fecal microbiota transplant (FMT) from healthy donors altered the microbiome composition in patients with metabolic syndrome and the coagulation system (Emdin et al., 2017). Although previous studies have suggested a link between gut microbiota, LEDVT, and PE, the genetic association between gut microbiota and LEDVT combined with PE has been largely understudied.

MR is a method that combines data from GWAS to infer causality by minimizing the influence of confounding factors such as the environment (Emdin et al., 2017). By utilizing MR methods, the present study sought to clarify the relationship between the gut microbiota and LEDVT, as well as LEDVT combined with PE, providing new evidence for further research in this field and building upon the reliability of previous MR studies in assessing causality.

## 2 Materials and methods

### 2.1 Exposure data

Genetic variants for human gut microbiota were taken from the latest GWAS summary data from the MiBioGen Consortium (2022) (Kurilshikov et al., 2021). This comprehensive investigation encompassed 18,340 individuals from 24 cohorts from the USA, Canada, Israel, South Korea, Germany, Denmark, the Netherlands, Belgium, Sweden, Finland, and the UK. The primary objective was to examine the relationship between autosomal human genetic variants and the gut microbiome, with a particular emphasis on characterizing the gut microbial composition at the genus level. Advanced taxonomic classification methods and Microbiota quantitative trait loci (mbQTL) mapping analysis were employed to identify host genetic variants associated with the abundance levels of bacterial taxa within the gut microbiota. Ultimately, a total of 211 taxa were identified, comprising 131 genera, 35 families, 20 orders, 16 classes, and 9 phyla (Kurilshikov et al., 2021).

### 2.2 Outcome data

Summary-level data of LEDVT and LEDVT combined with PE were retrieved from the IEU Open GWAS project: https://gwas.mrcieu.ac.uk/ (v7.5.3-2023-07-19, *N* = 42,350). Data for LEDVT (2,116 cases and 359,078 controls), LEDVT combined with PE (4,319 cases and 356,875 controls) were acquired from the UK Biobank (UKB) of European ancestry (Sudlow et al., 2015). The details are presented in Table 1.

**Table 1:** Characteristics of data sources and strength of IVs used in this study.

Trait	Consortium	Ethnicity	N. cases	N. controls	Number of SNPs
LEDVT	UK Biobank	European	2,116	359,078	10,544,982
LEDVT + PE	UK Biobank	European	4,319	356,875	11,783,033

LEDVT, lower extremity deep vein thrombosis; PE, pulmonary embolism; SNPs, single nucleotide polymorphisms; IVs, instrumental variables.

### 2.3 Instrumental variables selection

**Figure 1:**
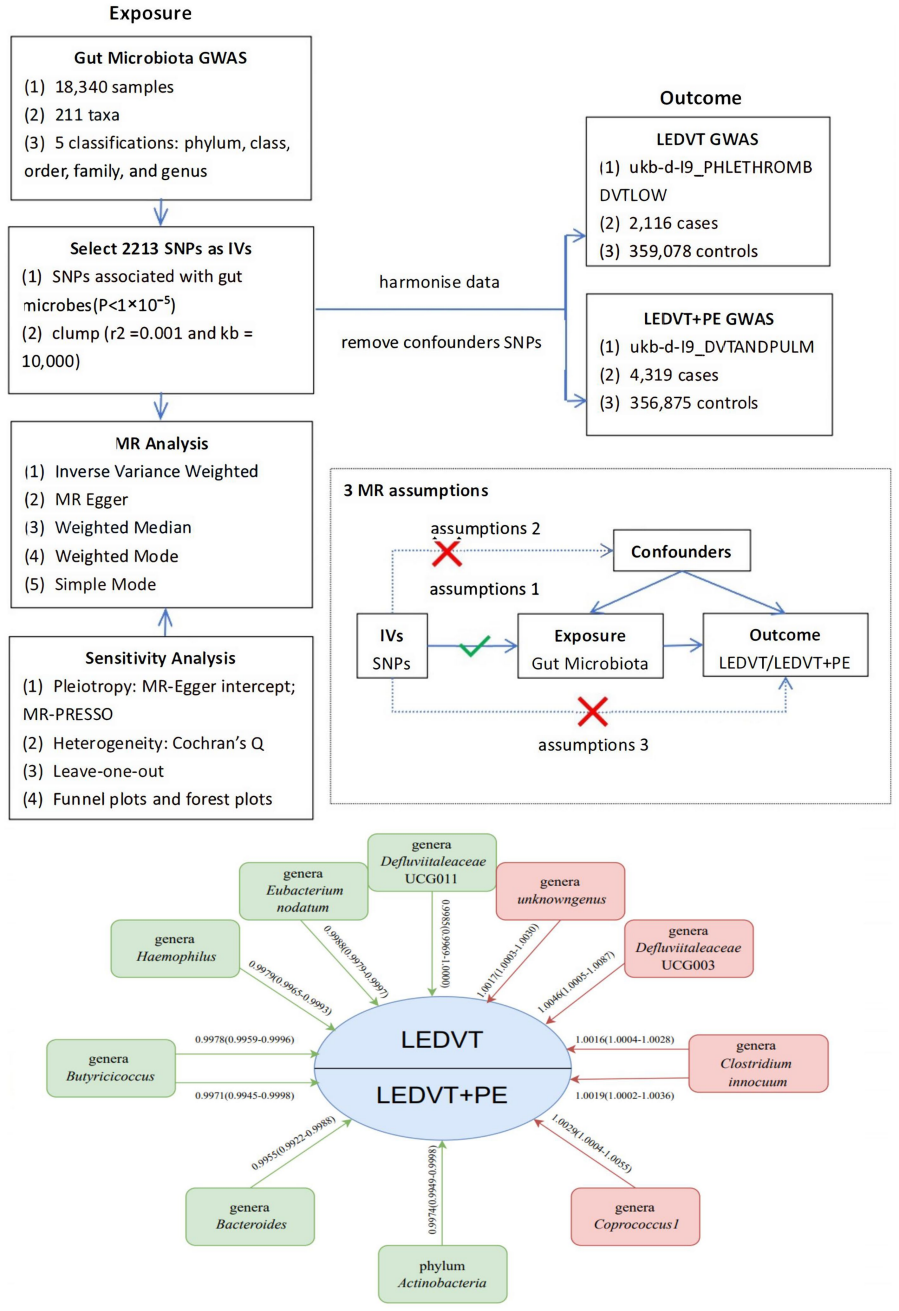
Overview of MR analyses process, major assumptions and major outcomes. GWAS, genome wide association study; SNPs, single nucleotide polymorphisms; IVs, instrumental variables; MR, Mendelian randomization; LEDVT, lower extremity deep vein thrombosis; PE, pulmonary embolism.

The study flowchart is illustrated in Figure 1. The exposure variable was the gut microbiota, while the outcome variable was LEDVT and LEDVT combined with PE. To enhance the validity and reliability of our findings, we used specific selection criteria for the identification of IVs: (1) single nucleotide polymorphisms (SNPs) associated with gut microbes were obtained with a significance threshold of (*p* < 1 × 10^−5^) (Sanna et al., 2019); (2) to account for the effect of linkage disequilibrium (LD), SNPs were clumped using an *r*^2^ threshold of 0.001 and a distance of 10,000 base pairs (Walker et al., 2019); (3) palindromic A/T or G/C alleles were excluded; (4) PhenoScanner^1^ was utilized to identify potential confounders (MR hypothesis II) by examining all SNPs with positive results (Kamat et al., 2019). The risk factors and confounders considered included smoking, obesity, hypercholesterolemia, hypertension, diabetes mellitus, Factor V Leiden, cancer, and, for women only, the use of oral contraceptives, hormone-replacement therapy, and menopausal status (Konstantinides et al., 2020); (5) the strength of the IVs was assessed using the F statistic (F = β^2^/se^2^). IVs with an F statistic >10 were considered to have no significant weak instrumental bias (Burgess et al., 2011).

### 2.4 Statistical analysis

All statistical analyses were performed using R software (Version 4.2.2). MR analyses were performed using the TwosampleMR (version 0.5.6) (Hemani et al., 2017) and MR-PRESSO (version 1.0) (Verbanck et al., 2018) R packages. A *p* < 0.05 was statistically significant.

### 2.5 Mendelian randomization analysis

We performed a MR analysis to investigate the causal relationship between the gut microbiome and LEDVT or LEDVT combined with PE. To ensure reliable results, the MR analysis satisfied three assumptions, as illustrated in Figure 1: (1) The IVs included in the analysis were strongly correlated with the exposure (gut microbiome). (2) The IVs used were not associated with the selected confounding factors related to both the exposure and the outcome. (3) The IVs affected the outcome solely through the exposure, without any direct effect (Davey Smith and Hemani, 2014).

In the MR analysis, we utilized several methods to estimate the causal effects. The IVW method assumes that all SNPs are valid instruments and estimates the overall effect by calculating the inverse variance-weighted mean of the individual instrument estimates. It has been reported that the IVW method has greater statistical efficiency when there is no horizontal pleiotropy bias (Burgess et al., 2013). In contrast, the MR-Egger method allows for some degree of pleiotropy, meaning that the resulting effect remains even when the intercept (effect of the instrument on the outcome when the exposure is zero) is zero (Bowden et al., 2015). The weighted median approach can yield reliable estimates even when it accommodates invalid instruments, such as in cases where 50% of the IVs are considered invalid (Bowden et al., 2016). Although the IVW method is commonly more effective than other methods in most cases, we also employed the MR-Egger, weighted median, weighted mode (Hartwig et al., 2017), and simple mode methods as supplementary analyses.

### 2.6 Sensitivity analysis

We conducted several sensitivity analyses to assess the robustness of our results. Cochran’s Q statistics were employed to assess the heterogeneity of the IVs. A *p*-value exceeding 0.05 suggested a lack of significant heterogeneity (Bowden and Holmes, 2019). We utilized the MR-Egger method to quantify the presence of pleiotropy in the IVs. A *p* > 0.05 for the MR-Egger intercept indicated the absence of horizontal pleiotropy (Burgess and Thompson, 2017). Acknowledging that MR-Egger regression may exhibit reduced accuracy and statistical power, we also implemented the MR pleiotropy residual sum and outlier (MR-PRESSO) method to identify any outliers that could suggest pleiotropic biases. A global test *p* > 0.05 in MR-PRESSO indicated no evidence of horizontal pleiotropy (Verbanck et al., 2018). The leave-one-out sensitivity analyses were conducted to assess the stability of results by excluding a single SNP each time.

## 3 Results

### 3.1 Instrumental variables selection

Initially, a total of 14,587 SNPs were identified as IVs from a GWAS focusing on the human gut microbiota, encompassing 211 bacterial traits categorized into five biological classifications: phylum, class, order, family, and genus (*p* < 1 × 10^−5^). To ensure the independence of the selected SNPs from the outcomes of interest (LEDVT and LEDVT combined with PE), we removed those SNPs with LD effects, yielding a final set of 2,213 SNPs as IVs. Comprehensive data regarding these SNPs, including the effect allele, alternative allele, beta coefficient, standard error (SE), and *p*-value, were systematically compiled for subsequent analysis.

### 3.2 Two-sample MR analysis

#### 3.2.1 Lower extremity deep vein thrombosis

**Figure 2:**
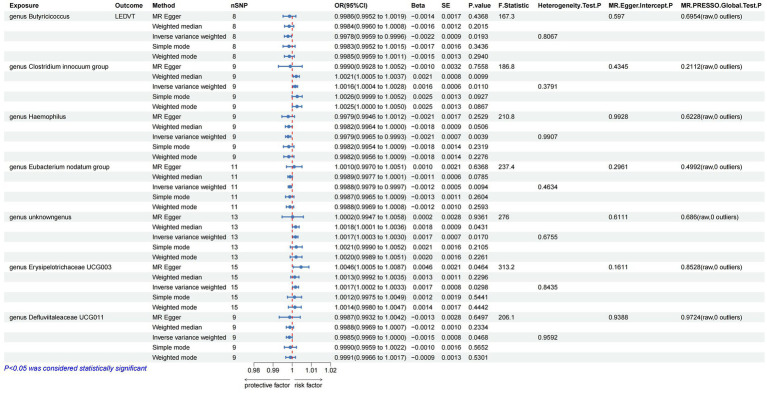
Mendelian randomization results of causal effects between gut microbiota and LEDVT (*P* < 1 × 10^–5^). LEDVT, lower extremity deep vein thrombosis; nSNPs, number of single nucleotide polymorphism; OR, odds ratio; CI, confidence interval; SE, standard error.

The results of IVW analyses suggested that the genera *Butyricicoccus* [odds ratio (OR) = 0.9978, 95% confidence interval (CI), 0.9959–0.9996, *p* = 0.0193], *Haemophilus* (OR = 0.9979, 95% CI, 0.9965–0.9993, *p* = 0.0039), *Eubacterium nodatum* (OR = 0.9988, 95% CI, 0.9979–0.9997, *p* = 0.0094), and *Defluviitaleaceae* UCG011 (OR = 0.9985, 95% CI, 0.9969–1.0000, *p* = 0.0468) were protective factors for LEDVT, while the genera *Clostridium innocuum* (OR = 1.0016, 95% CI, 1.0004–1.0028, *p* = 0.0110), *unknowngenus* (OR = 1.0017, 95% CI, 1.0003–1.0030, *p* = 0.0170), and *Erysipelotrichaceae* UCG003 (OR = 1.0046, 95% CI, 1.0005–1.0087, *p* = 0.0221) were risk factors for LEDVT (Figure 2).

#### 3.2.2 Lower extremity deep vein thrombosis combined with pulmonary embolism

**Figure 3:**
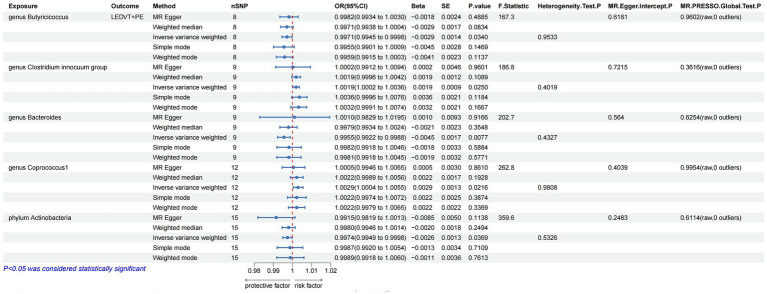
Mendelian randomization results of causal effects between gut microbiota and LEDVT + PE (*P* < 1 × 10^−5^). LEDVT, lower extremity deep vein thrombosis; PE, pulmonary embolism; nSNPs, number of single nucleotide polymorphism; OR, odds ratio; CI, confidence interval; SE, standard error.

The results of IVW analyses suggested that the genera *Butyricicoccus* (OR = 0.9971, 95% CI, 0.9945–0.9998, *p* = 0.0340) and *Bacteroides* (OR = 0.9955, 95% CI, 0.9922–0.9988, *p* = 0.0077), and the phylum *Actinobacteria* (OR = 0.9974, 95% CI, 0.9949–0.9998, *p* = 0.0369) were negatively correlated with LEDVT combined with PE risk, while the genera *Clostridium innocuum* (OR = 1.0019, 95% CI, 1.0002–1.0036, *p* = 0.0250) and *Coprococcus1* (OR = 1.0029, 95% CI, 1.0004–1.0055, *p* = 0.0216) were positively correlated with LEDVT combined with PE risk (Figure 3).

**Figure 4:**
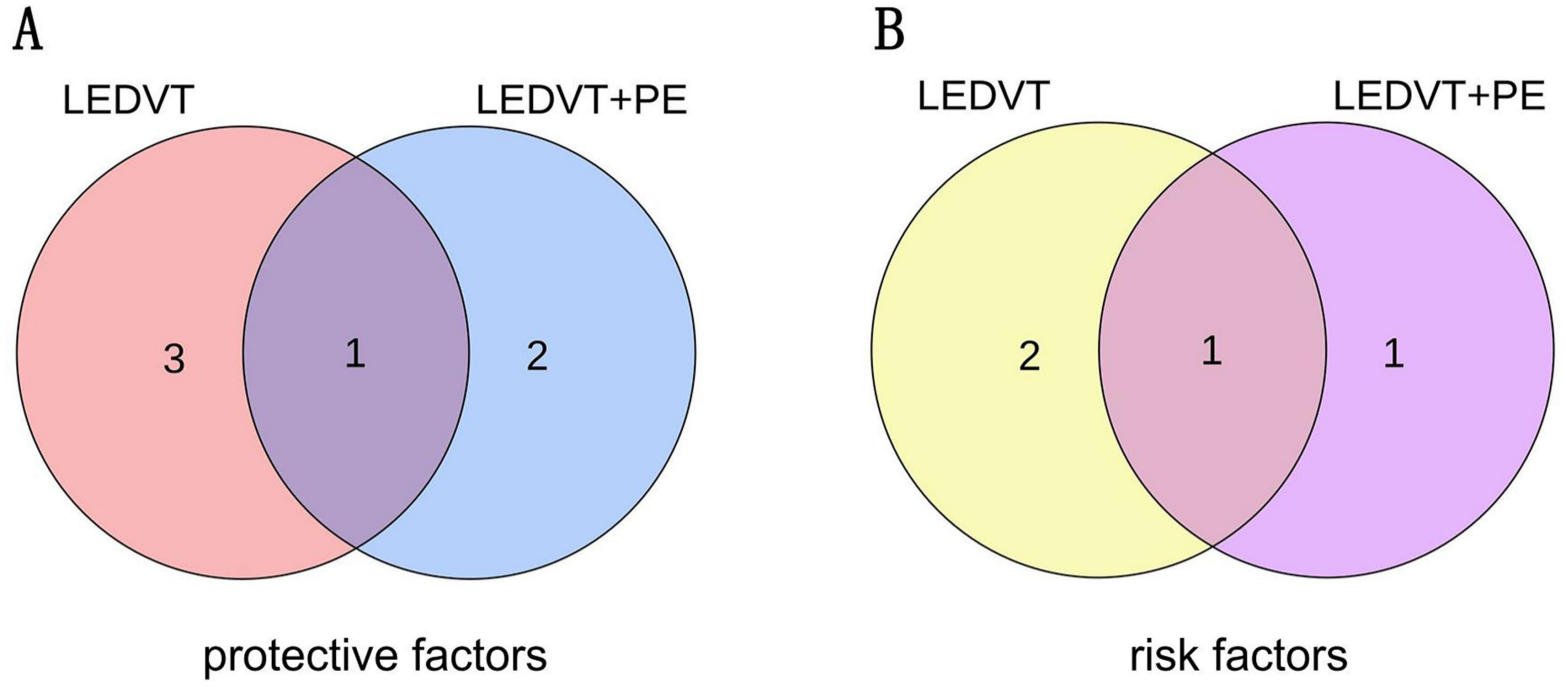
(A) The intersection of protective gut microbiota in LEDVT and LEDVT + PE GWAS. (B) The intersection of risk gut microbiota in LEDVT and LEDVT + PE GWAS. LEDVT, lower extremity deep vein thrombosis; PE, pulmonary embolism; GWAS, genome wide association study.

**Figure 5:**
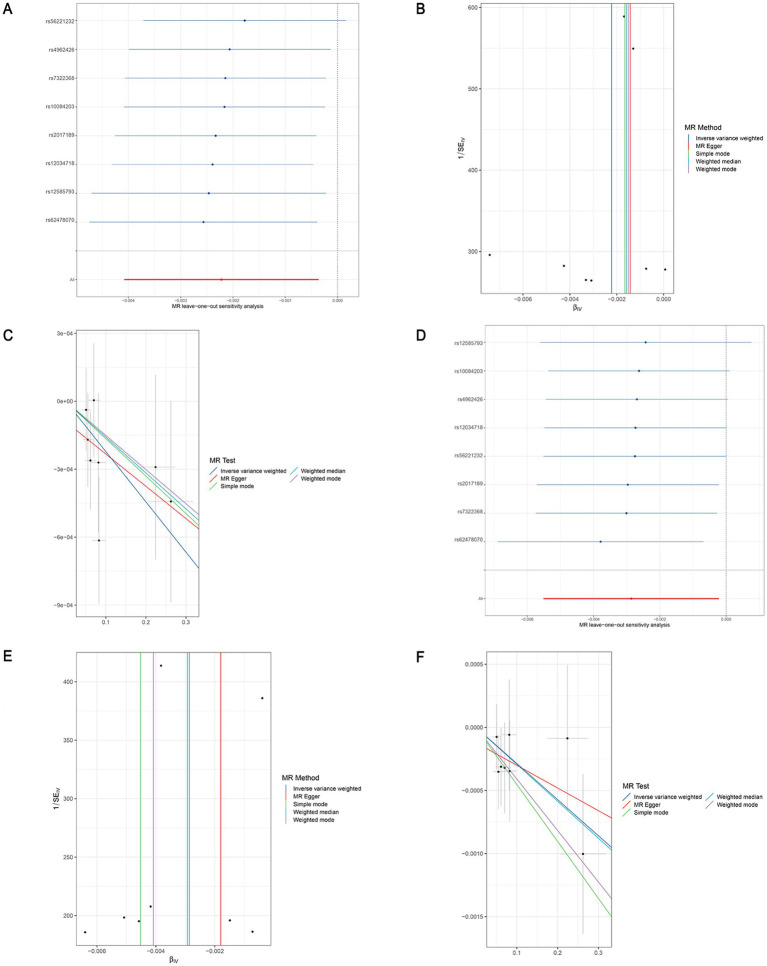
Leave-one-out sensitivity analyses (A), funnel plot (B), and scatter plot (C) of the causal effect of genus *Butyricicoccus* on LEDVT risk. Leave-one-out sensitivity analyses (D), funnel plot (E), and scatter plot (F) of the causal effect of genus *Butyricicoccus* on LEDVT + PE risk. LEDVT, lower extremity deep vein thrombosis; PE, pulmonary embolism.

**Figure 6:**
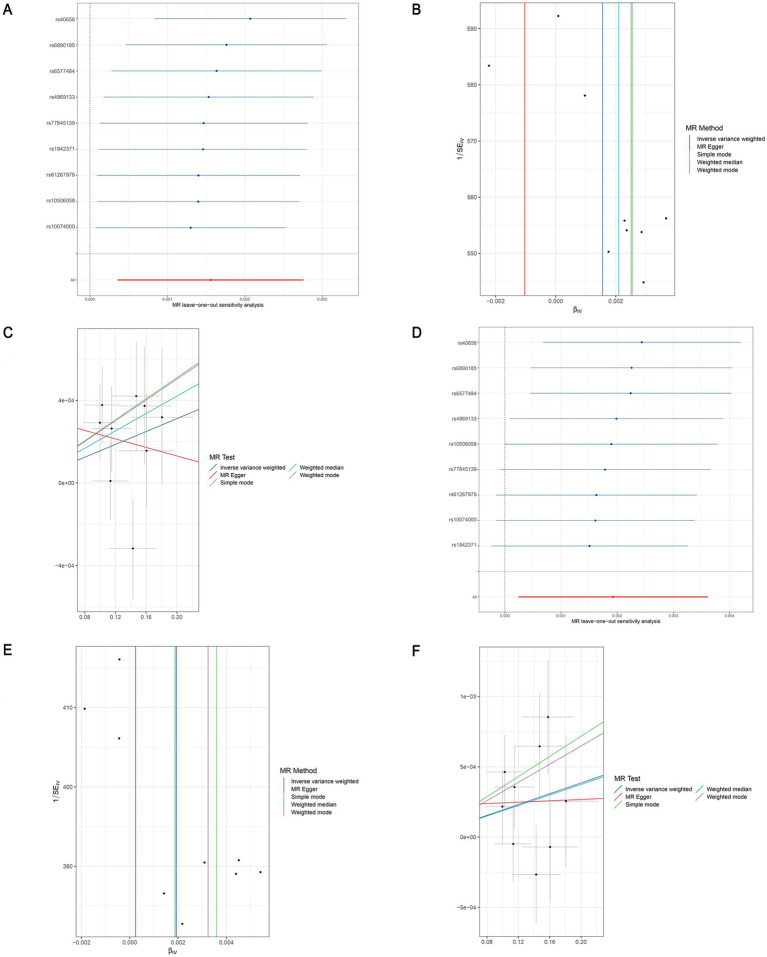
Leave-one-out sensitivity analyses (A), funnel plot (B), and scatter plot (C) of the causal effect of genus *Clostridium innocuum* group on LEDVT risk. Leave-one-out sensitivity analyses (D), funnel plot (E), and scatter plot (F) of the causal effect of genus *Clostridium innocuum* group on LEDVT + PE risk. LEDVT, lower extremity deep vein thrombosis; PE, pulmonary embolism.

After the two sets of results were intersected using a Venn plot (Figure 4), it was found that the genus *Butyricicoccus* reduced the risk of both LEDVT and LEDVT combined with PE (Figure 5), while the genus *Clostridium innocuum* increased the risk of both conditions (Figure 6).

### 3.3 Sensitivity analysis

In the analysis of causal associations, the F-statistics for the IVs exhibited a range from 167.331 to 359.589, indicating that weak IV bias was effectively mitigated (Figures 2, 3). The outcomes of Cochran’s IVW Q test indicated no significant heterogeneity among these IVs (*p* > 0.05) (Figures 2, 3). MR-Egger intercept test yielded a *p* > 0.05, suggesting the absence of horizontal pleiotropy (Figures 2, 3). Additionally, the MR-PRESSO global test confirmed the validity of the findings (Figures 2, 3). The leave-one-out analysis revealed that no individual SNP significantly influenced the MR estimation results (Supplementary Figure S1). Furthermore, the funnel plots (Supplementary Figure S2) demonstrated that all estimates adhered to the underlying assumptions, thereby reinforcing the credibility of the results.

### 3.4 Reverse MR

We performed MR Analysis to determine whether LEDVT or LEDVT combined with PE affects specific gut microbiota. We selected SNPs that were strongly associated with LEDVT or LEDVT combined with PE (*p* < 1 × 10^−5^, *r*^2^ = 0.001, kb = 10,000). The results of the Egger regression showed that there was no evidence of horizontal pleiotropy. Furthermore, the Cochran’s Q test indicated that there was no heterogeneity in the replication cohort, and all selected SNPs acted as strong IVs with F-statistics >10. The findings from the reverse MR analysis did not support causal relationships between the LEDVT or LEDVT combined with PE traits and the gut microbiota discussed earlier (Supplementary Table S1).

## 4 Discussion

In this study, we performed a two-sample MR analysis to evaluate the causal association between gut microbiota and LEDVT, as well as LEDVT combined with PE. Our findings revealed that certain genera, such as *Butyricicoccus*, *Haemophilus*, *Eubacterium nodatum*, and *Defluviitaleaceae* UCG011, were associated with a decreased risk of LEDVT. Conversely, genera like *Clostridium innocuum*, *unknowngenus*, and *Erysipelotrichaceae* UCG003 were identified as risk factors for LEDVT. Furthermore, genera including *Butyricicoccus*, *Bacteroides*, and phylum *Actinobacteria* showed a negative correlation with the risk of LEDVT combined with PE, while *Clostridium innocuum* and *Coprococcus1* were positively correlated with the risk of LEDVT combined with PE. To enhance the accuracy of the results, we intersected both sets of findings. Ultimately, our analysis indicated that the genus *Butyricicoccus* reduced the risk of both LEDVT and LEDVT combined with PE, whereas the genus *Clostridium innocuum* increased the risk of both conditions.

Among the gut microbiota that reduce the risk of LEDVT, the genus *Haemophilus* has been reported to be negatively associated with Multiple Sclerosis (Schepici et al., 2019) and decreased in patients with rheumatoid arthritis (Zhang et al., 2015), whereas a negative association with type 2 diabetes was found in a Systematic Review (Letchumanan et al., 2022). In an animal experiment, Heat-treated adzuki bean protein hydrolysates (APH) increased the genus *Eubacterium nodatum* to reduce cholesterol in mice (Zhao et al., 2022). In another report, the genus *Defluviitaleaceae* UCG011 was negatively associated with obesity in rats fed a high-fat diet (Zhang et al., 2021). Inflammation, diabetes, and hyperlipidemia are widely acknowledged as risk factors for LEDVT. Accordingly, we hypothesized that the reduction of LEDVT by these bacteria may be related to them. Considering that the genus Erysipelotrichaceae UCG003 has been linked to a heightened risk of inflammatory bowel disease in a previous study, it is plausible to suggest that it may also play a role in elevating the risk of LEDVT through the promotion of inflammatory processes.

Research reports indicate that in cases of weight loss due to obesity, the relative abundance of the genus *Bacteroides* tends to increase. Furthermore, when transferring the microbial communities of obese mice into germ-free mice, mice that receive the microbiota from obese mice gain more fat than those that receive microbiota from lean mice (Turnbaugh et al., 2006). It has been observed that *Bacteroides* can inhibit atherosclerosis by reducing the levels of LPS (Yoshida et al., 2018). The reduction of LPS, in turn, inhibits inflammation, lowers blood coagulation, and reduces platelet activity (Violi et al., 2023). A decrease in the phylum *Actinobacteria* has been associated with increased intestinal permeability, which allows LPS to enter the bloodstream. This leads to the activation of the immune system and the maintenance of chronic inflammatory states such as insulin resistance, diabetes, and liver disease (Binda et al., 2018). Moreover, the genus *Coprococcus1* can increase the risk of inflammatory bowel disease (Liu et al., 2022), and is related to high cholesterol (Morales et al., 2023), which increases the risk of obesity, which may account for the increased risk of LEDVT and PE (Wang et al., 2021).

In our study, the genus *Butyricicoccus*, mainly refers to the *Butyricicoccus pullicaecorum*, it is famous for producing butyric acid. It has been established that the genus *Butyricicoccus* is a gram-positive bacterium that is anaerobic and produces butyrate, found in the feces of healthy individuals. While no direct causal relationship has been established between *Butyricicoccus* and thrombosis, there are several reports indicating a negative correlation between *Butyricicoccus* and obesity, blood lipids (Zeng et al., 2019), and diabetes (Han et al., 2022). These factors suggest that *Butyricicoccus* may reduce the occurrence of coronary heart disease (Sun et al., 2022) and decrease liver injury by reducing the levels of LPS and inflammatory response (Li et al., 2023), which are high-risk factors for thrombosis patients, leading to the hypothesis that *Butyricicoccus* may reduce the likelihood of thrombus formation. The genus *Clostridium innocuum* is an anaerobic, Gram-positive, spore-forming bacterium first identified as a new species of the genus *Clostridium* by Smith and King in 1962 from a patient with an appendiceal abscess (Smith and King, 1962). Interestingly, the genus *Clostridium innocuum* has been positively associated with inflammatory bowel disease and increases the risk and prognosis of this patient population (Lunken et al., 2021). Additionally, it has been demonstrated to lead to severe infections, including endocarditis, osteomyelitis, peritonitis, and bacteremia (Cobo et al., 2023), and may promote adipogenesis, which may cause thrombosis (Ha et al., 2020). Nevertheless, further randomized controlled trials are needed to confirm these findings. In this study, the genera *Butyricicoccus* and *Clostridium innocuum* appeared in the two sets of GWAS data results, highlighting the accuracy and validity of this method, which is worthy of further verification and research.

The sample of our study was drawn from a large-scale multicenter database covering individuals of different ages, genders, and health status, which provides some support for the generality of the findings. However, it is important to note that the sample mainly comes from the specific geographical area and population, and other areas people may have different characteristics of the microbial community. Therefore, although our study had high internal validity, it should be taken with caution when generalized to other populations, and further validation in different contexts is needed.

In future studies, we will plan to conduct similar studies in different geographic regions and populations to verify the generalizability and applicability of our findings in other contexts. In addition, it is necessary to further study the biological mechanism between microbial genera and LEDVT and LEDVT combined with PE, and to use molecular biology and metabolomics technology to explore how microbial metabolites affect host physiological functions and cause thrombosis. Further, intervention studies can explore the potential of microbial genera in the prevention and treatment of VTE, such as evaluating the effect of dietary modification or probiotic intervention, and ultimately provide new strategies for clinical practice.

Nevertheless, some limitations of this study should be considered. First, in our GWAS data, all study populations were of European ancestry. Nonetheless, it should be borne in mind that disease patterns may differ between different ancestors, highlighting the need to interpret our conclusions with caution when extending our findings to other populations. Secondly, the analyses exhibited some heterogeneity, which can be attributed to the utilization of GWAS data for investigating potential nonlinear relationships and variations in age, health status, sex, and different layering effects. This heterogeneity could impact the results. Thirdly, it is important to note that potential pleiotropy cannot be completely ruled out, which may introduce biased estimates of causal effects. Fourthly, the low power in this study indicates that this may limit the statistical power of the study and suggests that future studies need larger sample sizes or stronger effect sizes to validate your study findings. Lastly, the analysis of bacterial taxa was limited to the genus level and did not consider more specialized levels, such as species or strain. By utilizing more advanced shotgun metagenomic sequencing in microbiota GWAS, more specific and accurate results can be obtained.

## 5 Conclusion

To summarize, our study revealed four positive causal directions and three negative causal directions with LEDVT, as well as three positive causal directions and two negative causal directions with LEDVT combined with PE. Notably, both the genera *Butyricicoccus* and *Clostridium innocuum* were present in these results, suggesting a closer association with the disease. These findings offer a new avenue for future research on the disease, which could have implications for its prediction, prevention, and treatment.

## Data Availability

Publicly available datasets were analyzed in this study. This data can be found at: https://mibiogen.gcc.rug.nl/; https://gwas.mrcieu.ac.uk/datasets/ukb-d-I9_DVTANDPULM/; https://gwas.mrcieu.ac.uk/datasets/ukb-d-I9_PHLETHROMBDVTLOW/.

## Footnotes

^1^http://www.phenoscanner.medschl.cam.ac.uk/
