# Supplementary material for: The causal relationship between gut microbiota and lower extremity deep vein thrombosis combined with pulmonary embolism
Source: Front Microbiol. 2024 Oct 2;15:1301737. doi: 10.3389/fmicb.2024.1301737 (PMC11480004; doi:10.3389/fmicb.2024.1301737)
Supplement: Supplementary file 1 [file Data_Sheet_1.pdf]

Supplementary materials

Figure S1 Leave-one-out sensitivity analysis of causal associations

A. Genus *Haemophilus* and Lower Extremity Deep Vein Thrombosis

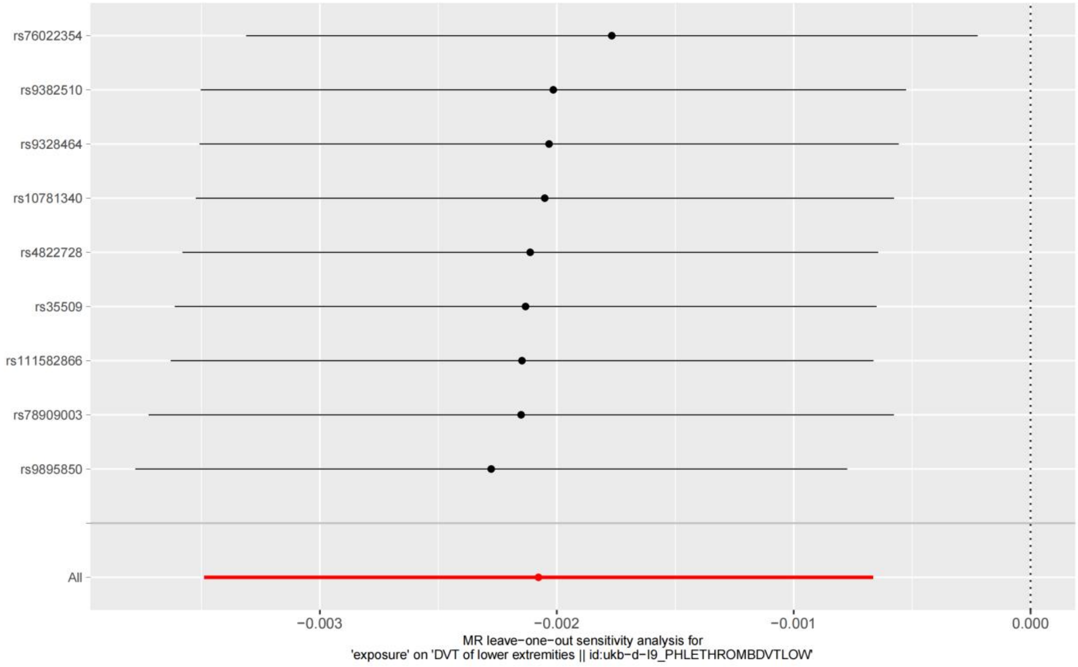


B. Genus *Eubacterium nodatum* group and Lower Extremity Deep Vein Thrombosis

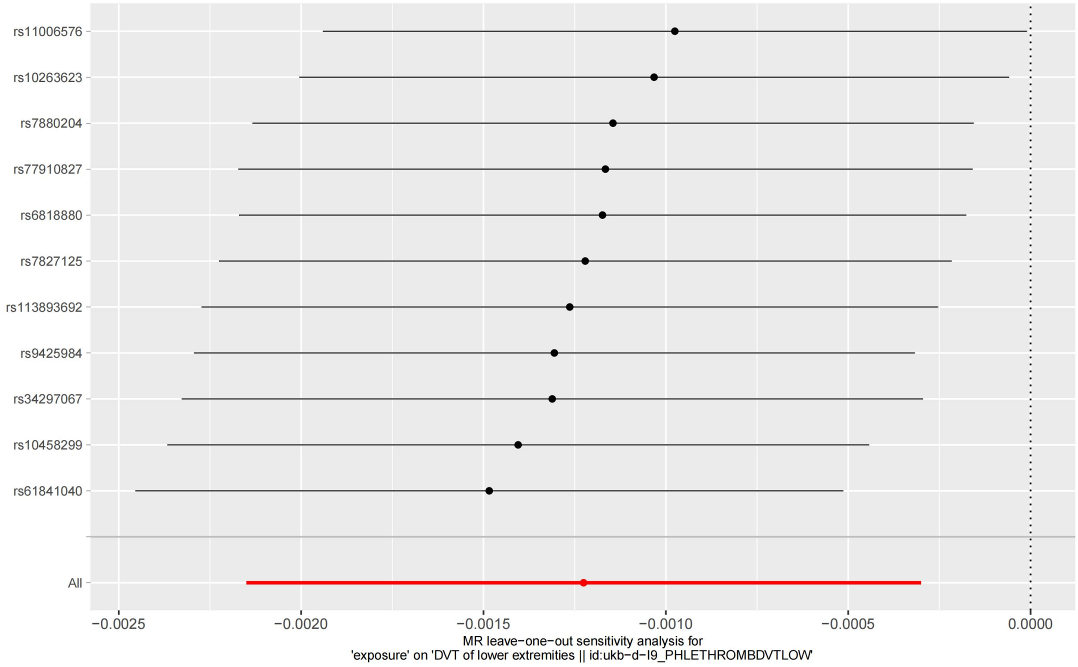

C. Genus *Defluviitaleaceae* UCG011 and Lower Extremity Deep Vein Thrombosis

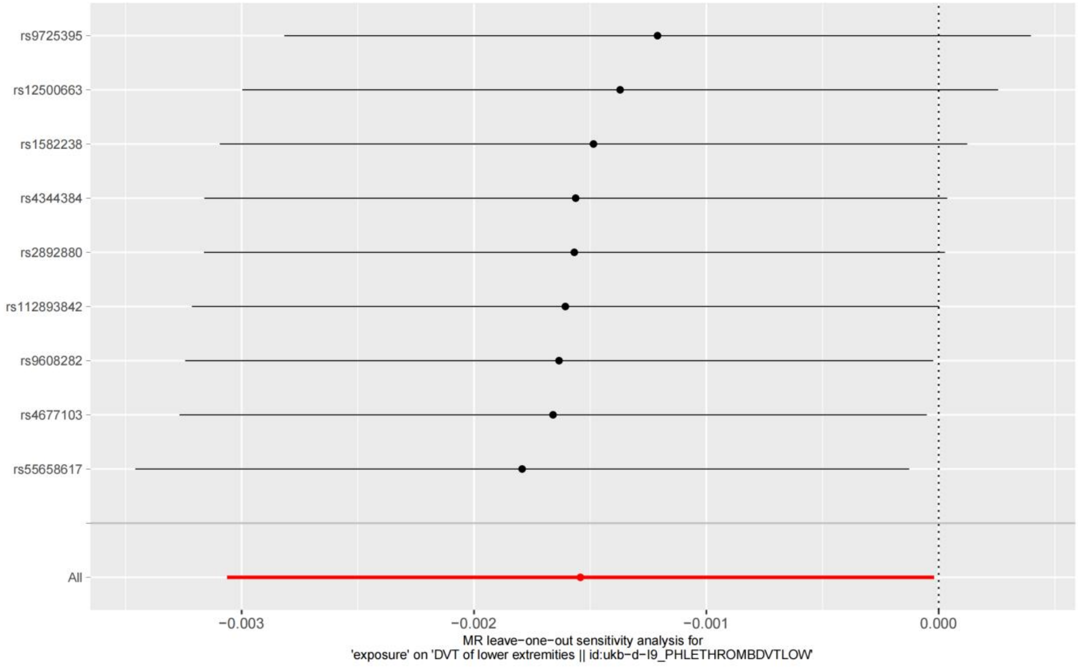


D. Genus *unknowngenus* and Lower Extremity Deep Vein Thrombosis

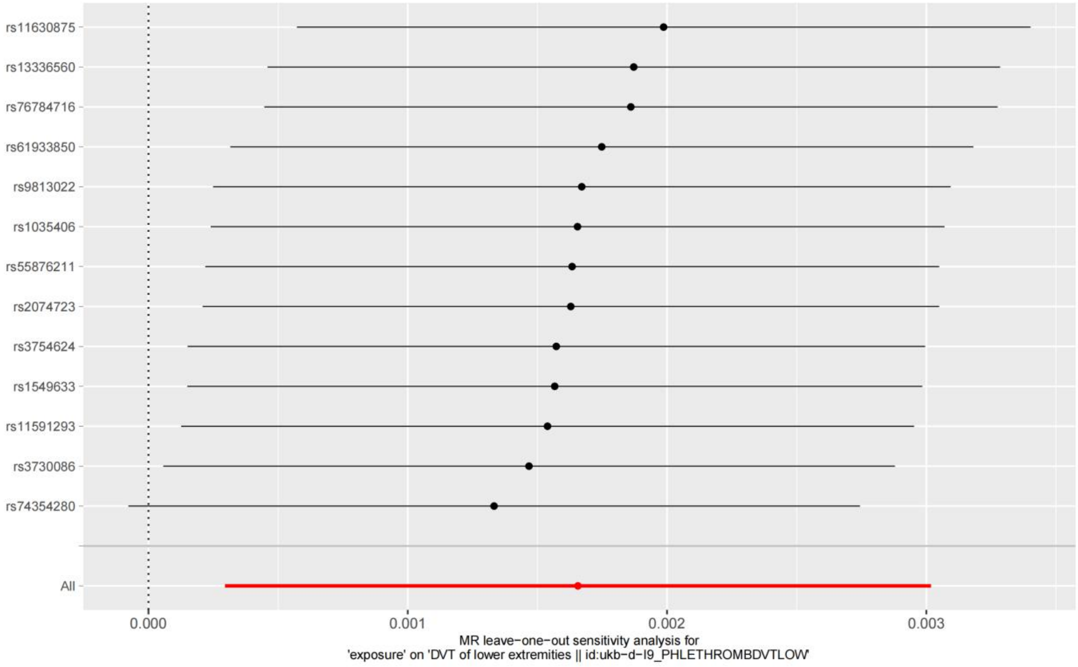

E. Genus *Erysipelotrichaceae* UCG003 and Lower Extremity Deep Vein Thrombosis

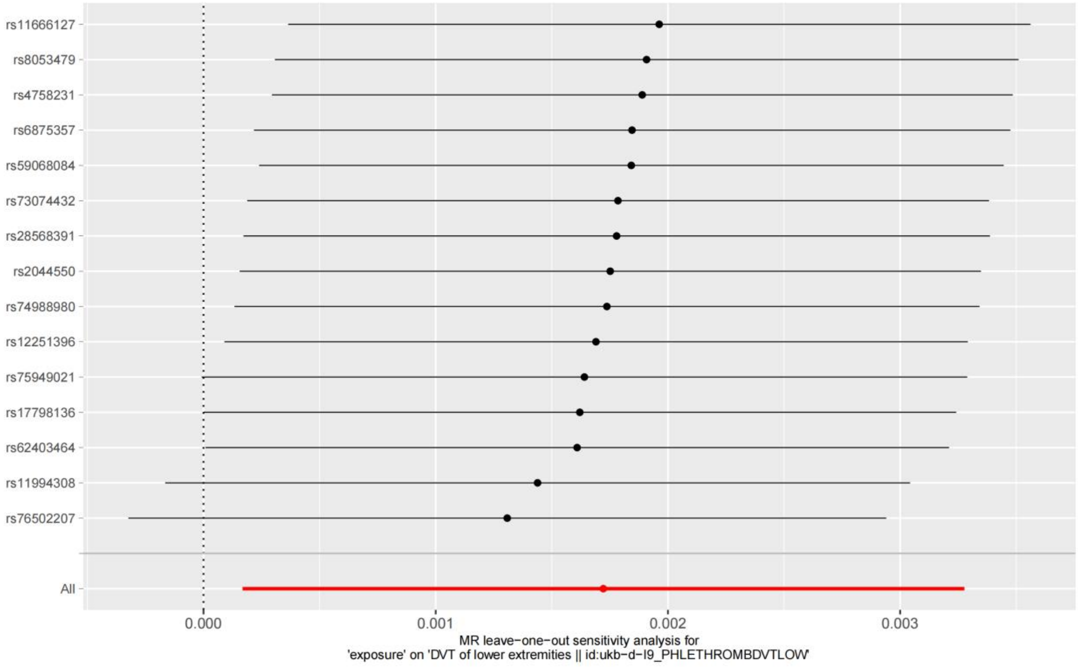


F. Genus *Bacteroides* and Lower extremity Deep Vein Thrombosis combined with Pulmonary Embolism

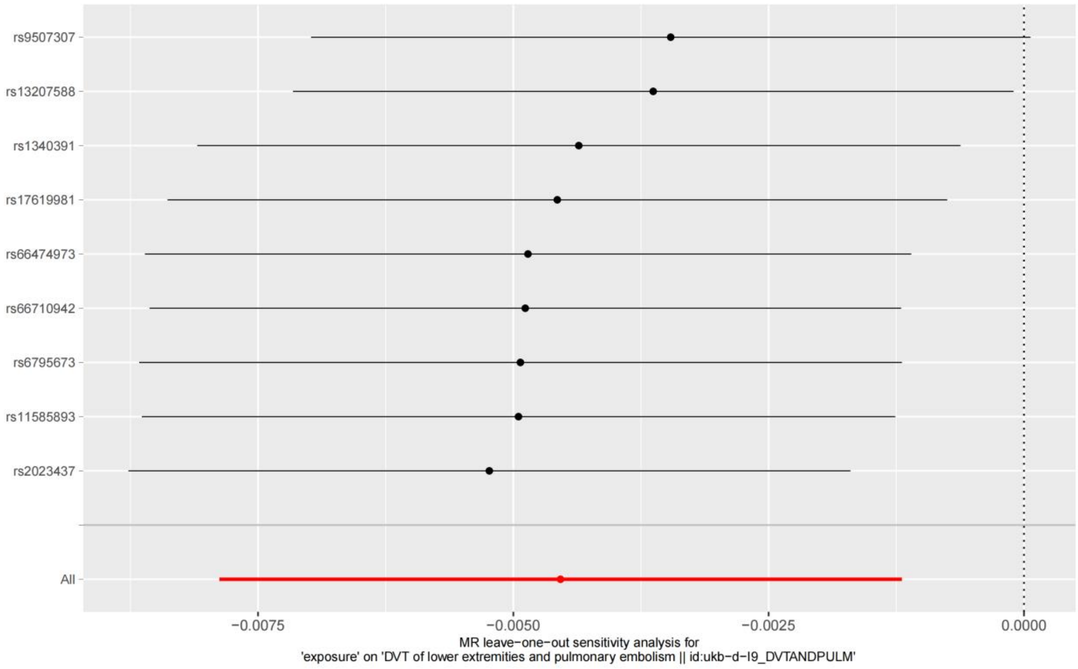

G. Phylum *Actinobacteria* and Lower extremity Deep Vein Thrombosis combined with Pulmonary Embolism

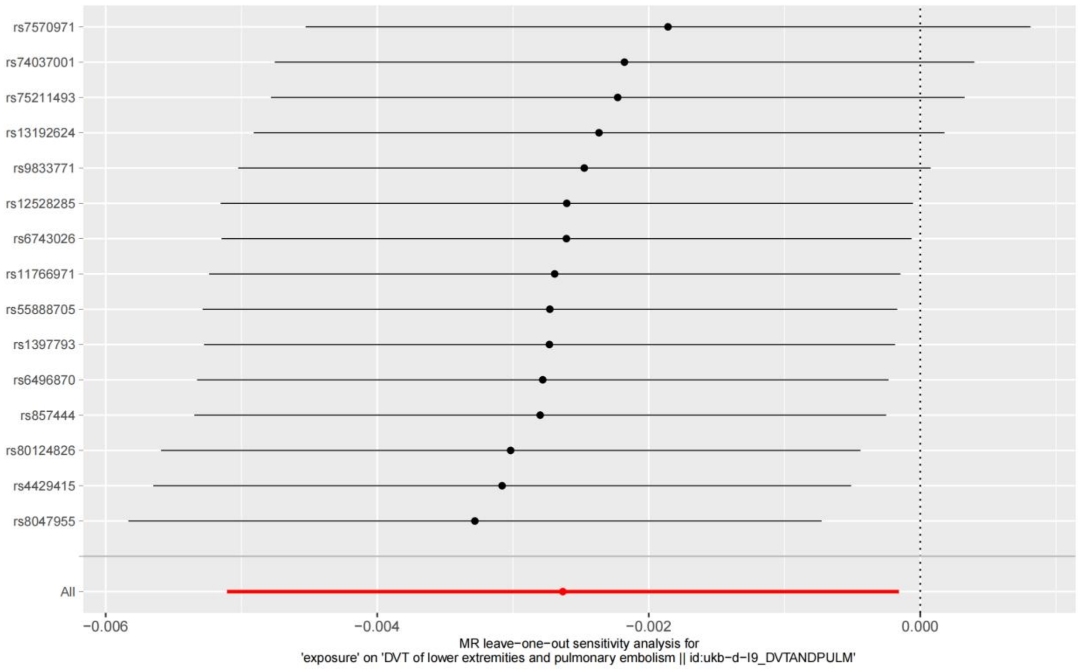


H. Genus *Coprococcus1* and Lower extremity Deep Vein Thrombosis combined with Pulmonary Embolism

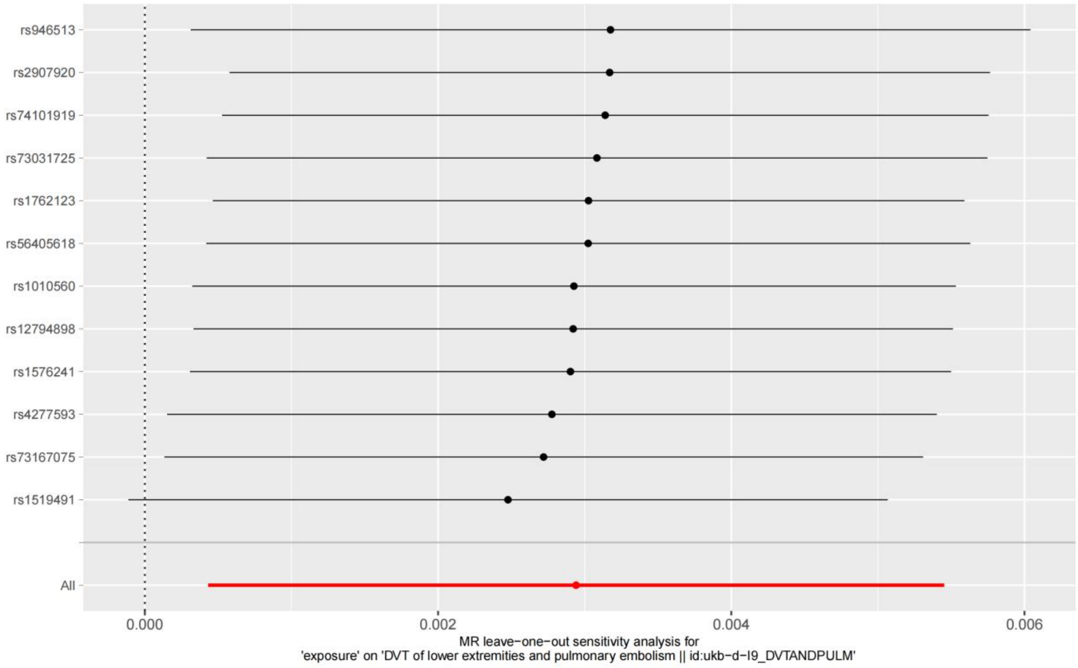

Figure S2 Funnel plot of causal associations

A. Genus *Haemophilus* and Lower Extremity Deep Vein Thrombosis

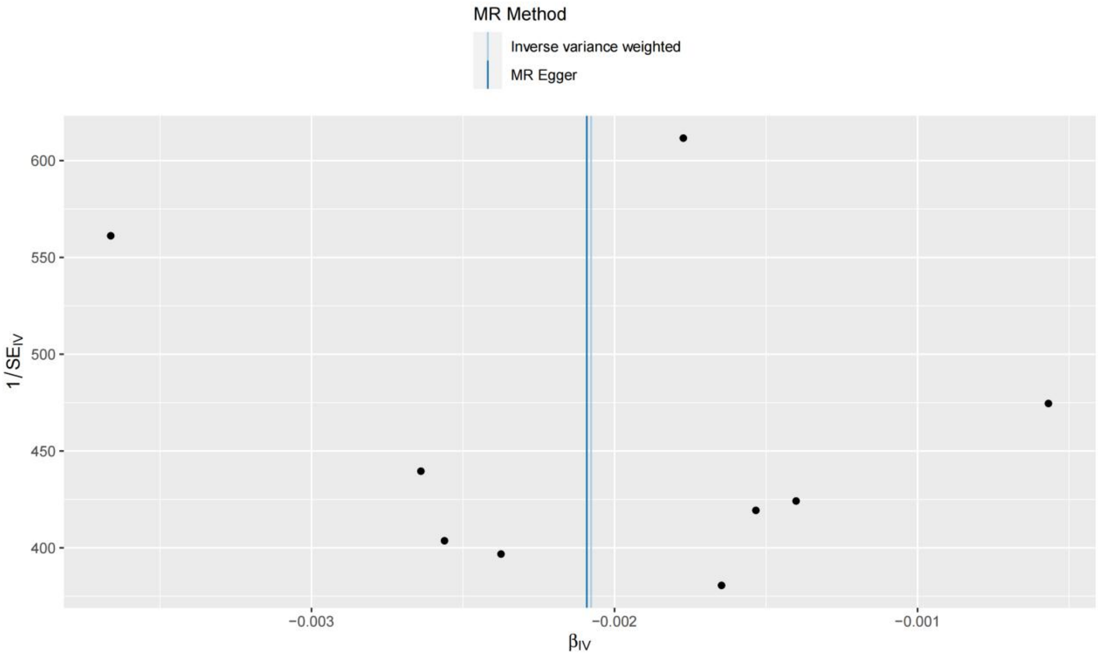


B. Genus *Eubacterium nodatum* group and Lower Extremity Deep Vein Thrombosis

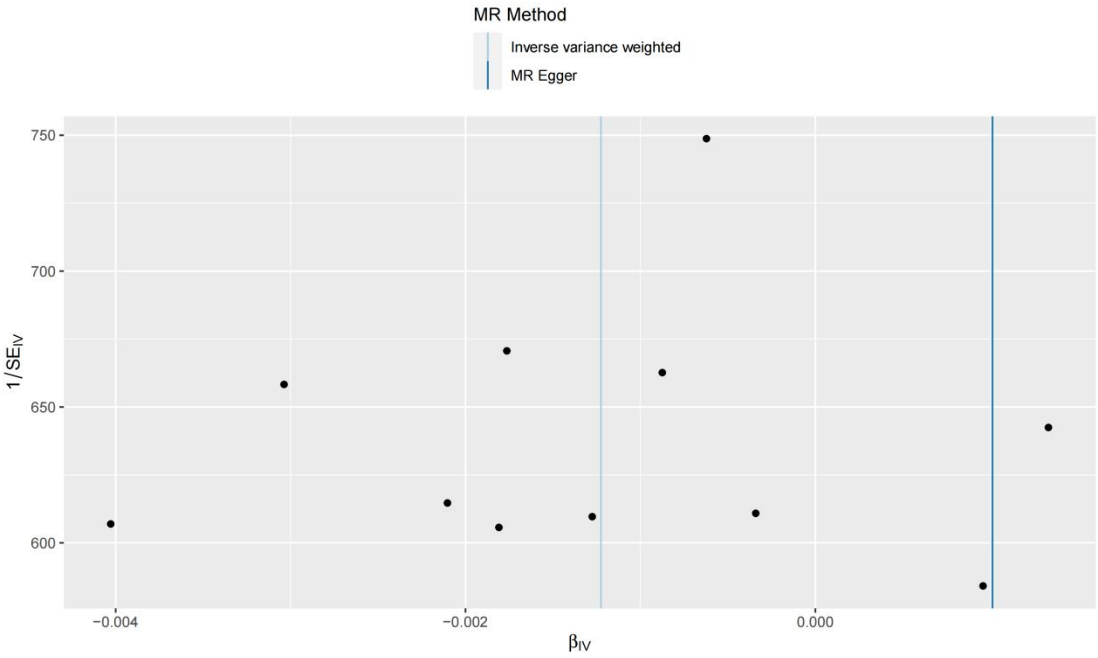

C. Genus *Defluviitaleaceae* UCG011 and Lower Extremity Deep Vein Thrombosis

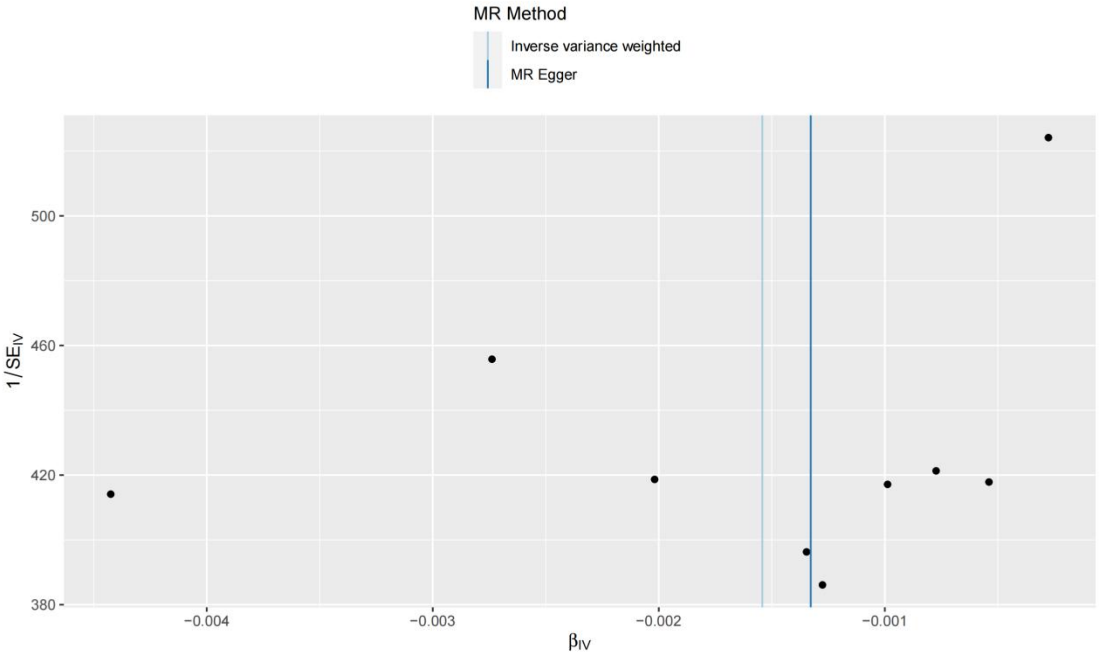


D. Genus *unknowngenus* and Lower Extremity Deep Vein Thrombosis

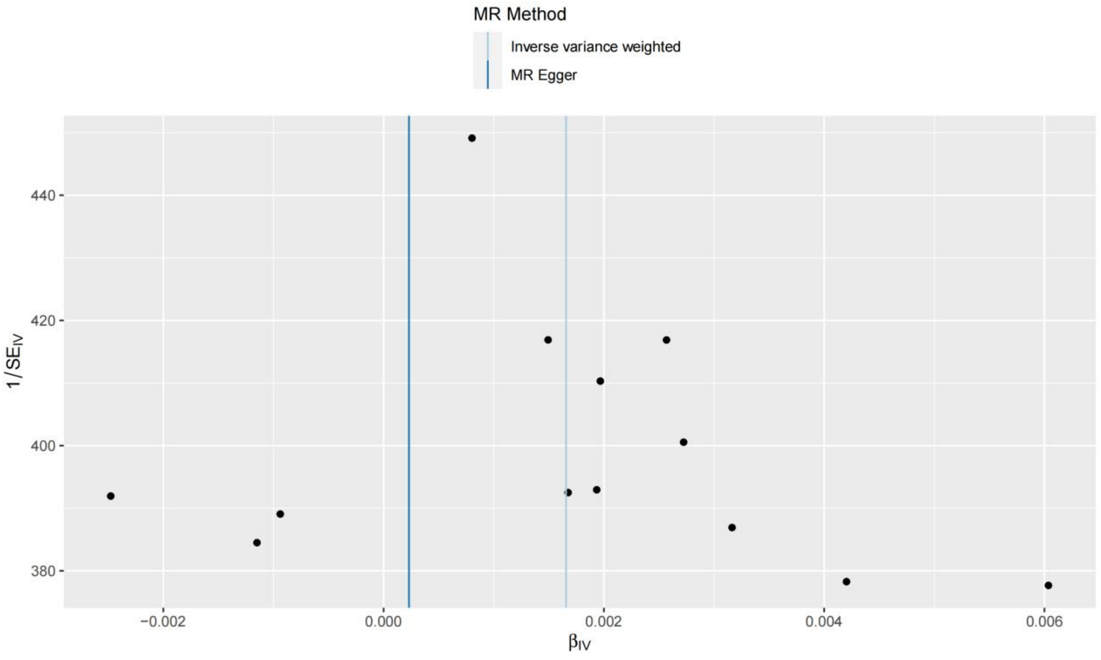

E. Genus *Erysipelotrichaceae* UCG003 and Lower Extremity Deep Vein Thrombosis

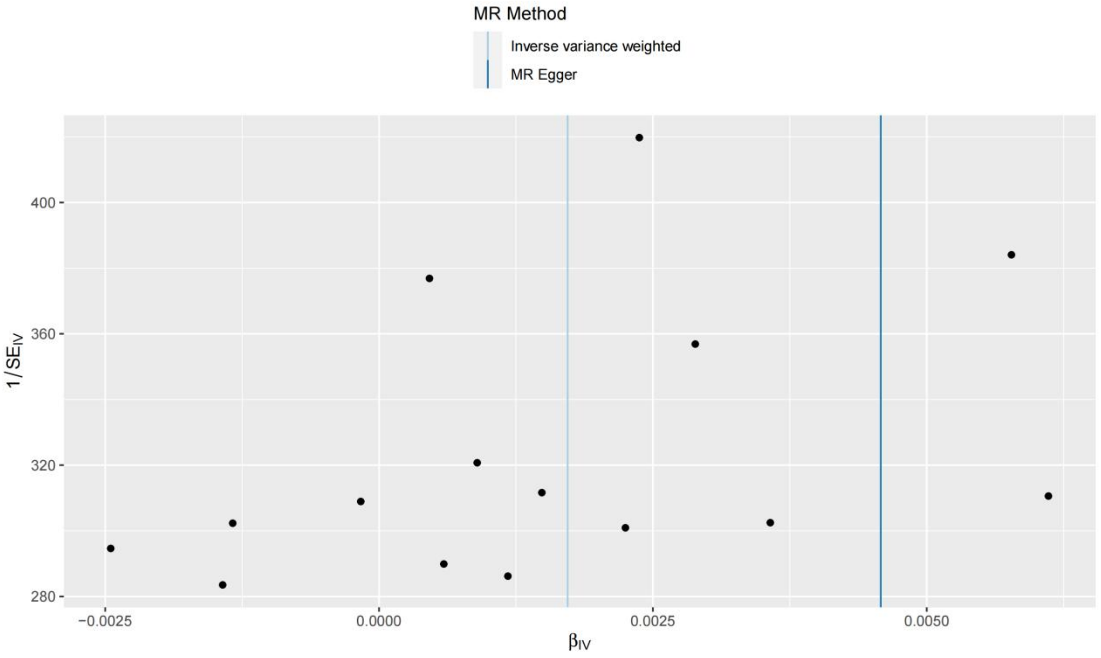


F. Genus *Bacteroides* and Lower extremity Deep Vein Thrombosis combined with Pulmonary Embolism

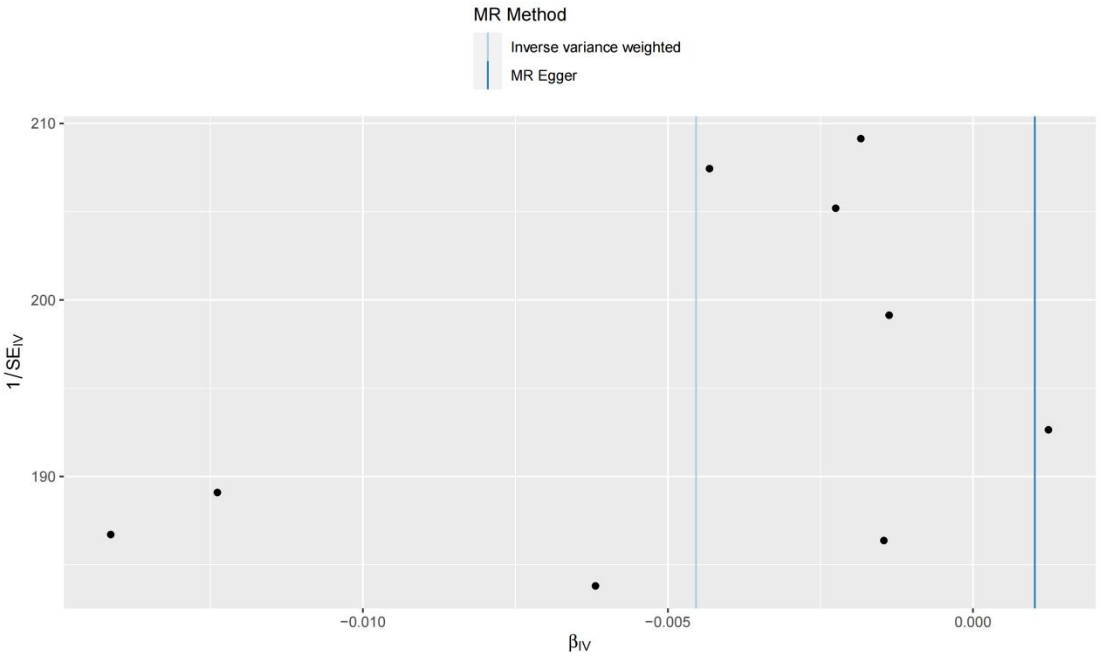

G. Phylum *Actinobacteria* and Lower extremity Deep Vein Thrombosis combined with Pulmonary Embolism

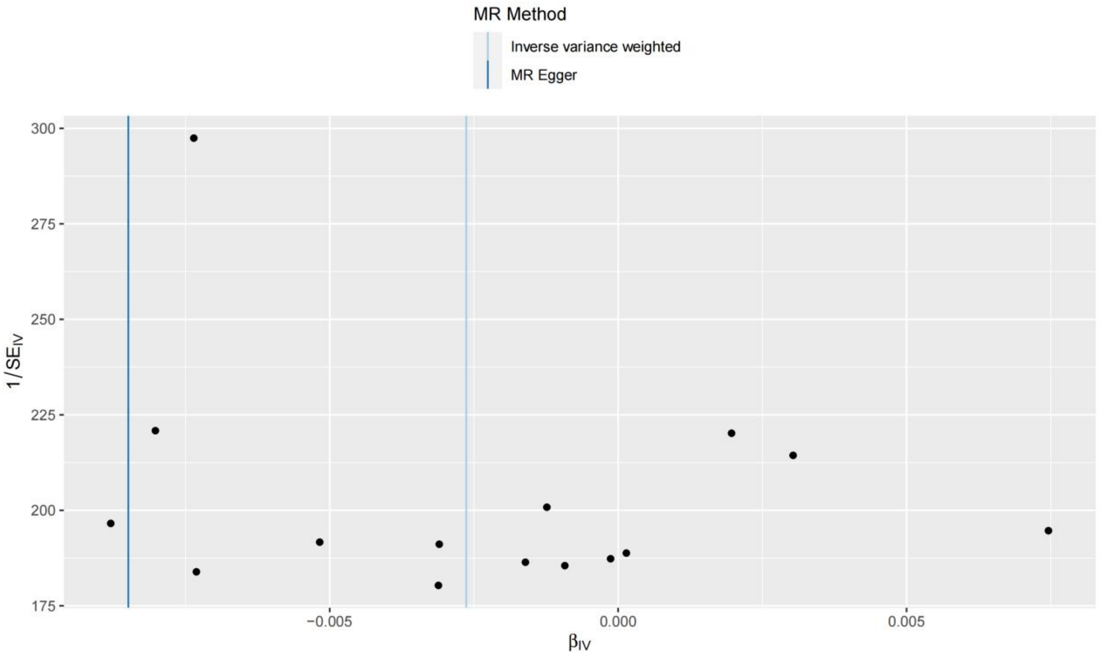


H. Genus *Coprococcus1* and Lower extremity Deep Vein Thrombosis combined with Pulmonary Embolism

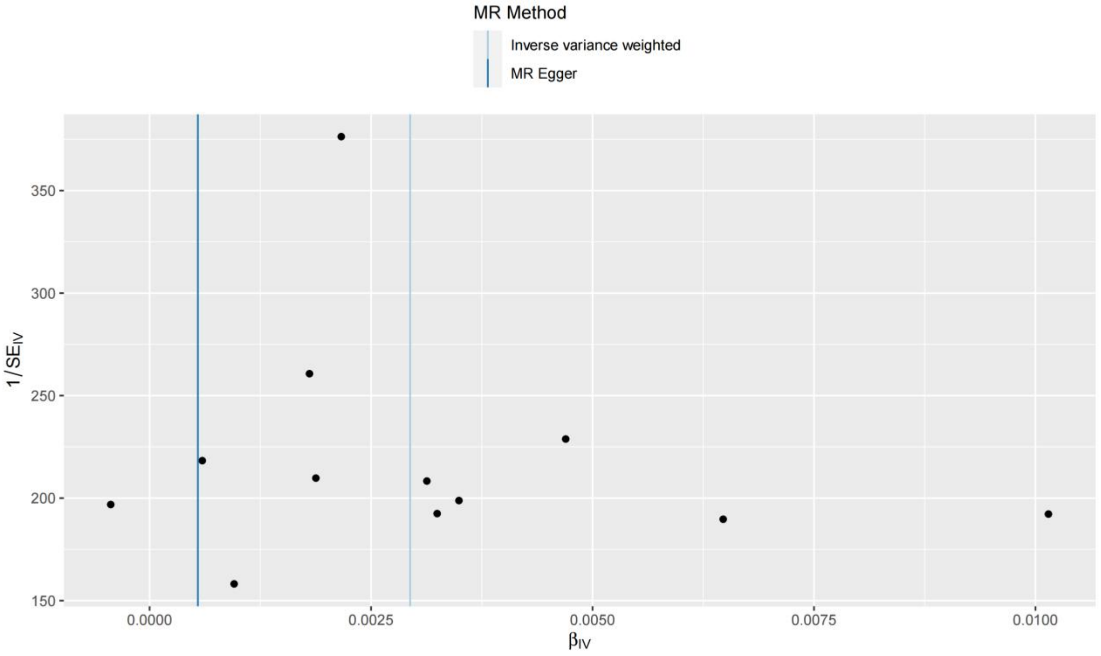

Table S1

MR Results for causal association of LEDVT/LEDVT+PE with gut microbiota

| Exposure | Outcome | Method | nSNP | Beta | SE | P. value |
|---|---|---|---|---|---|---|
| LEDVT | genus Allisonella | MR Egger | 18 | -11.2097 | 13.5250 | 0.4194 |
| | | Weighted median | 18 | -16.3341 | 7.8378 | 0.0372 |
| | | Inverse variance weighted | 18 | -16.0384 | 5.4457 | 0.0032 |
| | | Simple mode | 18 | -14.9654 | 12.8500 | 0.2603 |
| | | Weighted mode | 18 | -15.6225 | 8.6067 | 0.0872 |
| | family Peptococcaceae | MR Egger | 24 | 4.4158 | 7.2343 | 0.5479 |
| | | Weighted median | 24 | 4.0689 | 4.2461 | 0.3379 |
| | | Inverse variance weighted | 24 | 6.9143 | 2.7228 | 0.0111 |
| | | Simple mode | 24 | 11.6951 | 6.7496 | 0.0965 |
| | | Weighted mode | 24 | 4.3157 | 4.6401 | 0.3620 |
| | family Porphyromonadaceae | MR Egger | 24 | 11.4756 | 5.6057 | 0.0528 |
| | | Weighted median | 24 | 9.3093 | 2.9819 | 0.0018 |
| | | Inverse variance weighted | 24 | 4.8414 | 2.1060 | 0.0215 |
| | | Simple mode | 24 | 8.5245 | 5.7645 | 0.1528 |
| | | Weighted mode | 24 | 8.9237 | 3.5086 | 0.0182 |
| | genus Barnesiella | MR Egger | 24 | 5.8814 | 6.9628 | 0.4074 |
| | | Weighted median | 24 | 6.7837 | 3.7591 | 0.0711 |
| | | Inverse variance weighted | 24 | 5.7113 | 2.5587 | 0.0256 |
| | | Simple mode | 24 | 8.1388 | 6.3918 | 0.2156 |
| | | Weighted mode | 24 | 7.5474 | 4.2675 | 0.0902 |
| | genus Paraprevotella | MR Egger | 24 | 3.4627 | 9.7751 | 0.7265 |
| | | Weighted median | 24 | -4.5155 | 5.0197 | 0.3684 |
| | | Inverse variance weighted | 24 | -7.7494 | 3.7170 | 0.0371 |
| | | Simple mode | 24 | -17.5932 | 9.0295 | 0.0637 |
| | | Weighted mode | 24 | -4.9001 | 5.7800 | 0.4053 |
| LEDVT+PE | genus Eubacterium ventriosum group | MR Egger | 26 | -3.4002 | 2.6245 | 0.2074 |
| | | Weighted median | 26 | -3.2089 | 1.8274 | 0.0791 |
| | | Inverse variance weighted | 26 | -2.8397 | 1.2620 | 0.0244 |
| | | Simple mode | 26 | -2.1807 | 3.0036 | 0.4746 |
| | | Weighted mode | 26 | -3.2227 | 1.8679 | 0.0968 |
| | genus Ruminiclostridium | MR Egger | 26 | -0.5277 | 2.4626 | 0.8321 |
| | | Weighted median | 26 | -3.1660 | 1.8128 | 0.0807 |
| | | Inverse variance weighted | 26 | -2.6591 | 1.1834 | 0.0246 |
| | | Simple mode | 26 | -3.8690 | 3.3601 | 0.2604 |
| | | Weighted mode | 26 | -3.2429 | 2.1225 | 0.1391 |
| | genus Parabacteroides | MR Egger | 26 | 3.9520 | 2.4727 | 0.1231 |
| | | Weighted median | 26 | 3.8777 | 1.7139 | 0.0237 |
| | | Inverse variance weighted | 26 | 2.5731 | 1.1879 | 0.0303 |
| | | Simple mode | 26 | 3.2670 | 3.0246 | 0.2904 |
| | | Weighted mode | 26 | 3.6318 | 1.7156 | 0.0444 |

| | | | | | |
|---|---|---|---|---|---|
| genus Holdemanella | MR Egger | 25 | 8.3273 | 3.7127 | 0.0348 |
| | Weighted median | 25 | 6.1179 | 2.6410 | 0.0205 |
| | Inverse variance weighted | 25 | 3.5180 | 1.7874 | 0.0490 |
| | Simple mode | 25 | 6.1461 | 3.9972 | 0.1372 |
| | Weighted mode | 25 | 6.1461 | 2.7222 | 0.0333 |

MR, mendelian randomization; LEDVT, lower extremity deep vein thrombosis; PE,pulmonary embolism; nSNPs, number of single nucleotide polymorphism; SE, standard error.
